# Phytochemical Profiling and Computational Docking Studies Revealing the Potential Anticancer and Antioxidant Properties of *Heliotropium indicum* L.

**DOI:** 10.1155/sci5/1079290

**Published:** 2026-02-04

**Authors:** Sadia Afroz Shoily, Mst. Shahnaj Parvin, Mohsin Kazi, Aqibul Hasan Aqib, Md. Sabbir Hossain, Rasel Ahmed, Rafat Hossain Rafi, Jaytirmoy Barmon, Mohammad N. Uddin, Md. Ekramul Islam

**Affiliations:** ^1^ Department of Pharmacy, Faculty of Science, University of Rajshahi, Rajshahi, 6205, Bangladesh, ru.ac.bd; ^2^ Department of Pharmacy, Atish Dipankar University of Science and Technology, Dhaka, Bangladesh; ^3^ Department of Pharmaceutics, College of Pharmacy, King Saud University, P.O. Box 2457, Riyadh, 11451, Saudi Arabia, ksu.edu.sa; ^4^ Bangladesh Council of Scientific and Industrial Research, Rajshahi, 6206, Bangladesh, bcsir.gov.bd; ^5^ Department of Pharmaceutical Sciences, College of Pharmacy, Mercer University, Atlanta, Georgia, 30341, USA, mercer.edu

**Keywords:** Ehrlich ascites carcinoma, FTIR, GC‒MS analysis, molecular docking, PASS prediction, topoisomerase II inhibition

## Abstract

This study explores the anticancer and antioxidant potential of *Heliotropium indicum* L. through integrated phytochemical, biological, and computational approaches. GC–MS analysis identified phenol, 3,5‐bis(1,1‐dimethylethyl) as a major constituent. The chloroform fraction (CHF) exhibited significant *in vivo* antitumor activity in an Ehrlich ascites carcinoma (EAC) model, showing 76.39% tumor growth inhibition, comparable to bleomycin (80.36%). CHF also demonstrated potent cytotoxicity (LC_50_ = 28.96 μg/mL) in a brine shrimp assay and protected against AAPH‐induced oxidative DNA damage. Molecular docking revealed strong binding of key compounds to topoisomerase II, supported by stable molecular dynamics simulations and favorable MM–PBSA binding energies. These findings highlight *H. indicum* as a promising source of anticancer and antioxidant agents, warranting further mechanistic and preclinical investigation.

## 1. Introduction

Cancer remains one of the leading global health challenges, driving the need for safer and more effective therapeutic agents. Ehrlich ascites carcinoma (EAC) is widely used as an experimental model due to its high transplantability and rapid proliferation, making it suitable for evaluating antitumor and oxidative stress–related responses. Natural products continue to attract significant attention as potential anticancer sources because many plant‐derived compounds can suppress tumor growth while protecting normal cells from oxidative DNA damage.

Oxidative stress is a major contributor to carcinogenesis through the generation of reactive oxygen species (ROS), which induce base modifications, DNA strand breaks, and mutagenic lesions such as 8‐oxoguanine [1]. Although endogenous repair pathways counteract this damage, persistent oxidative injury disrupts genomic stability. Plant‐derived antioxidants—including phenolics, flavonoids, and related metabolites—play an essential role in neutralizing ROS and modulating pathways linked to inflammation, cell‐cycle progression, and apoptosis [2]. Several studies have reported that such phytochemicals exert selective cytotoxicity against tumor cells and can reduce tumor burden in EAC models [3].

*Heliotropium indicum* (Boraginaceae), commonly known as Indian heliotrope, has a longstanding use in traditional medicine for treating inflammation, wounds, infections, and various systemic ailments [4]. Previous phytochemical investigations have shown that *H. indicum* contains flavonoids, phenolic acids, terpenoids, and pyrrolizidine alkaloids (PAs). While some constituents demonstrate antioxidant and anti‐inflammatory effects, others particularly PAs pose potential hepatotoxic risks, underscoring the need for rigorous scientific evaluation [5]. Although several studies have explored selected bioactivities of *H. indicum*, its anticancer potential remains insufficiently characterized, especially using integrated experimental and computational approaches [5, 6].

Recent advances in computational biology, including molecular docking, Prediction of Activity Spectra for Substances (PASS) prediction, and long‐timescale molecular dynamics (MD) simulations, allow deeper mechanistic insight into how plant compounds interact with key cancer‐related targets such as topoisomerase II [7]. However, no previous study has combined *in vivo* antitumor evaluation, oxidative DNA protection assays, gas chromatography and mass spectrometry (GC–MS)‐based phytochemical profiling, PASS‐based activity prediction, validated docking, and μs‐scale MD simulation to identify and characterize potential anticancer compounds from *H. indicum*.

To address this gap, this study extends existing fraction‐based and *in silico*‐focused reports by integrating *in vivo* EAC antitumor evaluation with GC–MS profiling, validated docking, MD simulations, and molecular mechanics Poisson–Boltzmann surface area (MM–PBSA) analysis to provide a more comprehensive assessment of *H. indicum* bioactivity.

## 2. Materials and Methods

### 2.1. Collection and Preparation of Crude Plant Extract

The whole *Heliotropium indicum* plants were collected from rural agricultural margins in the Rajshahi district, Bangladesh (24.3745° N, 88.6042° E), during the flowering stage, when the plants were approximately 8–10 weeks old. Fresh specimens were brought to the Phytochemistry Laboratory, Department of Pharmacy, University of Rajshahi. Taxonomic identification was performed by an expert botanist in the Department of Botany, and a voucher specimen (No. DACB‐65969) was deposited in the University Herbarium for future reference. After collection, the plant materials were washed thoroughly with running tap water followed by distilled water to remove debris. The samples were then shade‐dried at 25°C ± 2°C in a well‐ventilated drying room using a hot air circulating drying chamber, avoiding direct sunlight to prevent degradation of heat‐ and light‐sensitive compounds. Drying continued for 10–12 days until constant weight was achieved.

Approximately 1 kg of the powdered material was extracted with methanol over 5 days. The extract was filtered, and the solvent was evaporated under reduced pressure at 40°C via a rotary evaporator, yielding a crude methanolic extract (CME). The methanol extract was fractionated using solvents with varying polarity, such as n‐hexane fraction (NHF), chloroform fraction (CHF), and ethyl acetate fraction (EAF). After fractionation, the samples were dried under reduced pressure in a rotary evaporator and stored in a refrigerator at 4°C for analytical purposes.

### 2.2. Fourier Transform Infrared (FTIR) Analysis

FTIR coupled with a universal attenuated total reflectance (ATR) allows the identification of functional groups in extracts [8]. The instrument (Model: L1600300, Spectrum Two, United Kingdom) with a DTGS detector was linked with PerkinElmer Spectrum IR software (Version 10.6.2). Spectra were recorded at a resolution of 5 cm^−1^ within the wavenumber range of 400–4000 cm^−1^ using a small drop of sample that was placed directly onto the diamond prism of the ATR attachment.

### 2.3. GC–MS

Sample preparation involved dissolving 20 mg of the extract in 2 mL of methanol (GC grade, Sigma‐Aldrich). The mixture was thoroughly homogenized via a vortex mixer to ensure uniformity and then filtered through a 0.2‐μm syringe filter to remove any solid particles. The filtered solution was transferred to a GC vial for subsequent GC‒MS analysis. GC‒MS analysis was conducted via a SHIMADZU GC‒MS QP‐2020 system equipped with an autosampler (AOC‐20s) and an autoinjector (AOC‐20i). An SH Rxi 5MS Sil column (30 m × 0.25 mm; 0.25 μm) [9] was used for separation. Helium was utilized as the carrier gas at a flow rate of 1.70 mL/min and a pressure of 115.8 kPa. The oven temperature program was as follows: an initial temperature of 80°C with a 2.0‐min hold, followed by a ramp of 7 C/min to 180°C with a 2.0‐min hold, and a final increase to 280°C with a 2.0‐min hold. A sample volume of 4.0 μL was injected in splitless mode with a split ratio of 50:1. Ionization was performed at 70 eV, and mass spectra were recorded over the range of 50–500 m/z during a total run time of 55 min. The solvent cutoff time was set to 3.5 min. For component identification, the acquired mass spectra were compared against the NIST08s, NIST08, and NIST14 libraries.

### 2.4. Brine Shrimp Cytotoxicity Assay

The cytotoxic activity was evaluated using the brine shrimp lethality assay [10]. The test samples were first dissolved in dimethyl sulfoxide (DMSO) and then further diluted with artificial seawater, ensuring that the final DMSO concentration did not exceed 0.05%. The total volume of each sample solution was adjusted to 5 mL with sea salt water. A 100 μL suspension containing 10 brine shrimp larvae (nauplii) was introduced into each test tube, followed by incubation for 24 h. After the incubation period, the number of dead nauplii in each tube was counted via a magnifying glass. The experiment included a vehicle‐treated control group along with multiple test concentrations, with each dose being tested in triplicate. Vincristine sulfate served as the positive control in all the experimental setups. Each concentration was tested in triplicate. Five concentrations (12.5, 25, 50, 100, and 200 μg/mL) were used to generate a dose–response curve. LC_50_ values were calculated using probit‐based nonlinear regression to ensure statistical reliability and reproducibility of the results.

### 2.5. *In Vivo* Antitumor Activity

#### 2.5.1. Animal Model

Swiss albino male mice weighing 25–30 g and aged 21–28 days were obtained from the animal house of the Department of Biochemistry and Molecular Biology, University of Rajshahi. Standard dry pellets and clean water were provided as feed, and they were housed under a 12 h day‒night cycle with a temperature of 22°C–28°C and a humidity of 55%. Approval of animal experiments was approved by the Institutional Animal, Medical Ethics, Bio‐Safety and Bio‐Security Committee (IAMEBBC), Institute of Biological Sciences, University of Rajshahi, Bangladesh, in accordance with standard guidelines for the care and use of laboratory animals. The use of human blood cells was also approved by the same committee.

#### 2.5.2. Tumor Model of the Experiment

The EAC tumors used in this study were obtained from the Department of Biochemistry and Molecular Biology at the University of Rajshahi, Bangladesh. Tumor aspiration was performed using a 5‐mL syringe equipped with a 20‐gauge needle. Freshly extracted tumor fluid was diluted with normal saline, and a hemocytometer was used to adjust the tumor cell count to approximately 3 × 10^6^ cells/mL. The viability of EAC cells was assessed via a 0.4% trypan blue dye exclusion assay, ensuring that only samples with over 90% viability were used for transplantation. Each mouse received an intraperitoneal injection of 0.1 mL of the prepared tumor suspension, with strict aseptic techniques maintained throughout the procedure [11, 12]. After tumor transplantation, mice were monitored daily for overall health, behavior, and signs of distress. Tumor growth was assessed indirectly by measuring body weight changes and abdominal distension. Any animal showing severe discomfort, lethargy, significant weight loss (> 15%–20%), or signs of distress was euthanized immediately, following humane endpoint guidelines. Water and food intake were observed regularly to ensure welfare standards. Control groups of mice were maintained in parallel, receiving equivalent volumes of normal saline intraperitoneally instead of the tumor suspension. These controls were monitored under the same conditions to differentiate the effects of tumor growth from normal physiological changes.

#### 2.5.3. Collection of EAC Cells

EAC cells were harvested from donor Swiss albino male mice weighing 25–30 g and suspended in sterile isotonic saline. Following the previously described methodology, a defined concentration of viable EAC cells (typically 1.5 × 10^6^ cells/mL) was introduced into the peritoneal cavity of each recipient mouse as a control. On the sixth day postimplantation, the mice were sacrificed, and the intraperitoneal tumor (EAC) cells were collected in normal saline.

#### 2.5.4. Antitumor Activity of the CHF

EAC is a rapidly proliferating and highly aggressive experimental tumor model widely used for evaluating antitumor efficacy *in vivo*. In EAC‐bearing mice, tumor progression is characterized by a marked increase in ascitic tumor cell count and white blood cells, accompanied by reductions in red blood cell count and hemoglobin levels due to tumor‐associated iron deficiency, hemolytic stress, or myelopathic effects. Effective anticancer agents are known to mitigate these tumor‐induced alterations and restore hematological parameters toward normal values [13, 14]. In the present study, the *in vivo* antitumor activity of the CHF was evaluated by assessing ascitic tumor cell growth, hematological parameters, and morphological changes of EAC cells [15]. Because EAC is a nonsolid, intraperitoneal tumor model, solid tumor volume measurement was not applicable; therefore, tumor progression was appropriately monitored using ascitic tumor cell counts, body weight changes, and abdominal distension, which are established and widely accepted endpoints for EAC models.

#### 2.5.5. Experimental Design

Swiss albino mice (*n* = 20) were randomly assigned to 4 groups of 5 animals each. Group I served as the healthy control and received only the vehicle solution without tumor cells. Group II was the experimental control, receiving 1 × 10^6^ EAC cells intraperitoneally and treated with vehicle. Group III received the same EAC inoculation and was treated with the test compound CHF at 5 mg/kg/day, a dose selected based on preliminary toxicity and efficacy studies (data are not shown here). Group IV, the standard treatment group, received bleomycin at 0.3 mg/kg/day as a reference chemotherapeutic agent.

EAC cells were propagated in donor mice and harvested 6–7 days post‐ascites development. Cells were counted using a hemocytometer, diluted to 1 × 10^6^ cells/0.1 mL in normal saline (0.9%), and assessed for viability (> 90%) via 0.4% trypan blue exclusion before injection. Tumor inoculation was performed intraperitoneally, and treatments began 24 h postinoculation for 6 consecutive days. Each injection was 0.1 mL per mouse. Mice were monitored daily for body weight, food and water intake, general health, and signs of distress, following established humane endpoints. Blinding was applied during tumor cell counting and data analysis to reduce bias. On Day 7, mice were sacrificed, and ascitic tumor cells were collected by peritoneal flushing with saline. Viable tumor cells were counted, and tumor growth inhibition was calculated as follows:

(1) % cell growth inhibition=1−TwCw×100,

where Tw represents the mean number of tumor cells in Groups III or IV and Cw represents the mean number of tumor cells in Group II.

#### 2.5.6. Assessment of Biochemical Parameters

At the end of the experimental period (12 days), overnight fasted rats were sacrificed, and blood was collected for hematological parameters such as albumin, serum glutamic oxaloacetic transaminase (SGOT), serum glutamic pyruvic transaminase (SGPT), and total protein. These parameters were measured via commercially available kits following previously described methods [16].

### 2.6. Evaluation of Antioxidant Activity Using DNA Damage Protection Assay

To assess the antioxidant potential of the extracts, a DNA damage protection assay was employed using oxidative stress induced by AAPH. Reaction mixtures were prepared by combining 100 μg/mL of EAF, CHF, CME, and ascorbic acid (used as a standard antioxidant), each from a 4 mg/mL stock solution. To each mixture, 10 μL of plasmid DNA was added in separate Eppendorf tubes. Controls included DNA alone (blank), DNA + AAPH (negative control), and DNA + ascorbic acid + AAPH (positive control). All treatments and controls were performed in triplicate. The oxidative reaction was initiated by adding 2 μL of AAPH (a peroxyl radical generator), and the final volume was adjusted to 10 μL with phosphate‐buffered saline (PBS). The mixtures were incubated at 37°C for 30 min to allow oxidative stress to occur. After incubation, 0.2 μL of 6X loading dye (in a 1:1 ratio) was added to each sample. The samples were then subjected to electrophoresis on a 1% agarose gel using 1X TAE buffer at 120 V for 15 min. Following electrophoresis, the gel was stained with ethidium bromide (EtBr) for 15–20 min, and the DNA bands were visualized under UV light. Images were captured using the Alpha Imager Red‐Protein Simple gel documentation system. Protection of DNA from oxidative damage was assessed by comparing the intensity and integrity of DNA bands among treated and untreated samples.

### 2.7. Computational Analysis

#### 2.7.1. *In Silico* PASS Prediction Study

The online tool PASS (https://www.pharmaexpert.ru/passonline/) was employed to assess the biological activity spectra of key components in *H. indicum*. The structures of these components were sourced from PubChem and subsequently converted into SMILES format. The results of the PASS calculations are represented as Pa (probability of the molecule being active) and Pi (probability of the molecule being inactive), with both scores ranging from 0.00 to −1.00. If Pa exceeds 0.7, the substance has a high likelihood of exhibiting activity in experiments, but there is also a significant chance that it resembles a known pharmaceutical agent. If Pa is between 0.5 and 0.7, the substance is likely to show activity in experiments, albeit with a lower probability, and it is less similar to existing pharmaceutical agents. If Pa is less than 0.5, the substance is unlikely to demonstrate activity in experiments. However, if such activity is confirmed, the substance could be identified as a new chemical entity.

### 2.8. Computational Methods

#### 2.8.1. Ligand and Protein Preparation

Several phytocompounds identified in *Heliotropium* indicum extracts via GC–MS analysis were selected for *in silico* evaluation. Their two‐dimensional (2D) structures were retrieved from the PubChem database and converted into three‐dimensional (3D) PDB format using Open Babel. These ligands were then energy‐minimized using the universal force field (UFF) and assigned Gasteiger charges through PyRx 0.8.

Target proteins, including topoisomerase II, were downloaded from the RCSB Protein Data Bank (PDB). The proteins were refined using BIOVIA Discovery Studio (v21.1), including removal of water molecules and heteroatoms, correction of missing residues, and addition of polar hydrogen atoms. The prepared protein structures were saved in PDBQT format for molecular docking.

#### 2.8.2. Molecular Docking Protocol

Molecular docking simulations were conducted using AutoDock Vina implemented within the PyRx suite. A rigid docking protocol was applied to the proteins, while ligands were kept flexible. The docking grid box was centered on the active site of the protein with coordinates (*X* = 45.2, *Y* = 32.8, *Z* = 15.6) and dimensions of 20 × 20 × 20 Å. Docking exhaustiveness was set to 8. Redocking and ligand docking were performed using identical grid box coordinates and exhaustiveness parameters, ensuring methodological consistency and reliable RMSD‐based validation. The top‐ranked poses were selected based on binding energy scores (kcal/mol) and further analyzed using Discovery Studio for hydrogen bonding, hydrophobic interactions, and binding pocket residues.

#### 2.8.3. Virtual Screening and Interaction Visualization

Prior to docking, all ligands were optimized and converted to PDBQT format. Protein–ligand interactions were visualized using Discovery Studio Visualizer. Amino acid residues involved in binding and types of interactions (e.g., hydrogen bonding, π–π stacking, hydrophobic) were recorded for each complex.

#### 2.8.4. MD Simulations

MD simulations were carried out to assess the stability and dynamic behavior of the ligand–topoisomerase II complexes using GROMACS 2022. The AMBER99SB‐ILDN force field was applied for proteins and GAFF for ligands, with topologies generated via the ACPYPE server. Complexes were solvated in a TIP3P water box with 10 Å spacing and neutralized with counterions. Energy minimization was conducted using the steepest descent algorithm (5000 steps), followed by two equilibration phases: 100 ps under NVT (constant volume) and 100 ps under NPT (constant pressure) using the V‐rescale thermostat (300 K) and Parrinello–Rahman barostat (1 bar). The production phase ran for 150 ns with a 2‐fs time step and trajectory recording every 10 ps. Structural stability was assessed through RMSD, RMSF, radius of gyration (Rg), solvent‐accessible surface area (SASA), and hydrogen bond analysis.

#### 2.8.5. Binding Free Energy Calculation

Binding free energy was estimated using the MM/PBSA approach, applied to selected frames from the 150‐ns MD trajectories. The calculations were performed using the g_mmpbsa tool in GROMACS. Contributions from electrostatic, van der Waals, polar solvation, and nonpolar solvation energies were considered.

#### 2.8.6. Ligand Efficiency (LE) and Ki Estimation

To provide a deeper understanding of the binding efficiency and drug‐likeness of the key phytocompounds, LE and estimated inhibition constant (Ki) values were calculated using binding energy data from molecular docking. LE was calculated using the following formula:

(2) LE=–ΔGN_heavy_atoms ,

where Δ*G* is the docking binding energy (in kcal/mol) and N_heavy_atoms is the number of nonhydrogen atoms in the molecule.

Inhibition constant (Ki) was estimated using the following formula:

(3) Ki=expΔG×10001.987×T, where T=298 K.

### 2.9. Statistical Analysis

Statistical analyses were conducted via IBM SPSS Statistics for Windows, Version 27.0.1 (SPSS Inc., Chicago, IL) and Microsoft Excel 2021. To assess differences among sample means, ordinary one‐way analysis of variance (ANOVA) was performed, followed by Tukey’s honestly significant difference (HSD) test.

## 3. Results

### 3.1. Functional Groups Identified by FTIR Analysis

**FIGURE 1:**
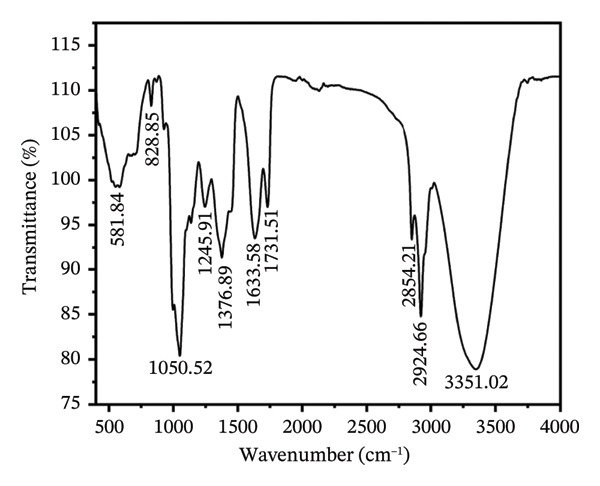
Characterization of functional groups in the methanolic extract of *H. indicum* by FTIR–ATR spectroscopy.

FTIR is highly helpful for characterizing the constituents present in plant extracts since it reveals the presence of both organic and inorganic compounds in plants. The biological or therapeutic activity of *H. indicum* is indicated by the presence of functional groups in its leaves. FTIR was used to assess the potential contribution of the methanolic extract to the identification of the chemical compositions of the organic components. The hydroxyl stretching vibration in close proximity to 3351.02 cm^−1^ is indicated by strong, wide absorption in the methanolic extract of this plant, as shown in Figure 1. This absorption is caused by the molecular interactions between the chains of polysaccharides. In the same spectral region, it was also observed that the N‐H bonds in the amino groups were stretched symmetrically and asymmetrically, which suggests the possibility that alkaloids may be the result of N‐H stretching [17, 18]. The extract’s symmetric and asymmetric CH_2_ stretching vibrations originated from the two sharp bands at 2924.66 and 2854.21 cm^−1^, respectively. The signal recorded at 1731.51 cm^−1^ is consistent with the presence of carbonyl stretching vibrations. The signal detected at 1633.58 cm^−1^ is consistent with flavonoids, amino acids, a twisted aromatic ring, and the C=C group’s stretching vibrations. The C‐H deformation vibrations of the aromatic ring and alkane‐, alkanol‐, and amine‐type compounds are indicated by the peak at 1376.89 cm^−1^. The mid‐infrared absorption bands at 1245.91 and 1050.52 cm^−1^ were mostly attributed to C‐O stretching vibrations in glycosidic bonds and pyranoid rings, although their structures and compositions varied, indicating the existence of polysaccharides as the primary component [11]. The measurement range for bending vibration is 800–500 cm^−1^. Table 1 lists all the functional groups that are involved.

**TABLE 1:** Wavenumber of responsible functional groups in the methanolic extract of *H. indicum* determined via FTIR–ATR analysis.

Serial number	Frequency (cm^−1^)	Responsible functional group	Types of compounds
1	3351.02	O‐H stretching	Alcohols, phenols, water
2	2924.66	C‐H asym. stretching	Alkanes
3	2854.21	C‐H sym. stretching	Alkanes
4	1731.51	C=O stretching	Carbonyl compounds (ketones, aldehydes, carboxylic acids)
5	1633.58	C=C stretching	Alkenes, aromatics
6	1376.89	C‐H deformation vibrations	Alkane, alkanol, amine
7	1245.91	C‐O stretching	Esters, ethers, alcohols
8	1050.52	C‐O stretching	Esters, ethers, alcohols
9	828.85	Bending vibrations	Various bending vibrations, aromatics
10	581.84	Bending vibrations	Various bending vibrations, aromatics

### 3.2. GC–MS Analysis

**FIGURE 2:**
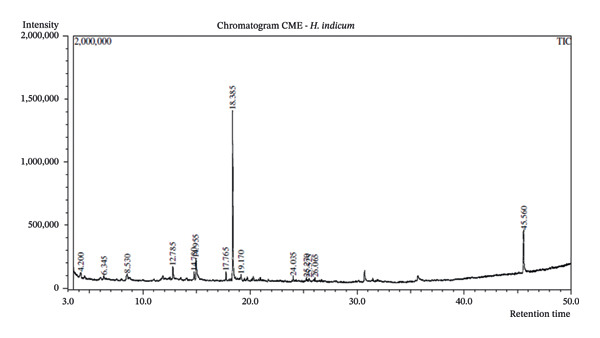
GC‒MS spectra of the crude methanolic extract (CME) of *H. indicum*.

In the present study, GC‒MS analysis was conducted on the CME of *H. indicum* to identify and predict the total number of bioactive compounds present. This analytical approach allowed for the detailed characterization of the chemical constituents, providing insights into their potential bioactivities. The results of the GC‒MS analysis are visually represented in Figure 2, which displays the corresponding GC‒MS spectra, illustrating the peaks associated with the detected compounds. The relative abundance of compounds was determined using % peak area, which provides semiquantitative estimation but not absolute concentration. Table 2 summarizes the key parameters for each identified compound, including its retention time, mass‐to‐charge ratio (m/z), relative peak area, and concentration, providing a comprehensive profile of the bioactive components in the extract.

**TABLE 2:** Compounds identified in the crude methanolic extract (CME) of *H. indicum* by GC‒MS analysis.

Compound name	Retention time (min)	Peak area	Concentration (%)	Mass‐to‐charge ratio (m/z)	Similarity index (SI)	Signal‐to‐noise ratio (S/N)
3‐Carene	4.196	24,689	1.27080	93.00	80	6.76
Benzoin methyl ether	6.341	45,554	2.34477	121.00	90	15.05
1‐Undecene	8.533	14,276	0.73482	55.00	78	2.37
9‐Methoxybicyclo[6.1.0]nona‐2,4,6‐triene	12.785	37,305	1.92017	115.00	96	11.94
9‐Octadecene, (E)‐	14.783	26,031	1.33988	55.00	93	9.01
Benzenemethanol, α‐methyl‐α‐propyl‐	14.955	45,429	2.33833	131.00	93	13.35
Tridecanol, 2‐ethyl‐2‐methyl‐	17.761	55,929	2.87879	57.00	96	12.10
Phenol, 3,5‐bis(1,1‐dimethylethyl)‐	18.387	1,345,025	69.23151	191.00	100	379.16
Dodecane, 2,6,11‐trimethyl‐	19.170	35,820	1.84374	71.00	93	3.05
Tridecanol, 2‐ethyl‐2‐methyl‐	24.032	26,247	1.35099	57.00	91	5.56
Eicosane	25.272	20,636	1.06218	57.00	92	4.34
1‐Octanol, 2‐butyl‐	25.521	16,329	0.84049	57.00	87	2.82
1‐Pentadecene, 2‐methyl‐	26.062	12,325	0.63440	57.00	89	2.74
Di‐n‐octyl phthalate	45.559	237,198	12.20912	149.00	99	65.66

### 3.3. Protection of Oxidative DNA Damage by CHF, EAF, and CME

**FIGURE 3:**
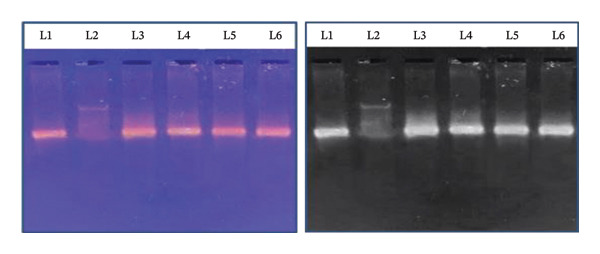
Protective effects of CHF, EAF, and CME of *H. indicum* L. extracts on DNA damage induced by AAPH. Lane 1: native DNA; Lane 2: DNA + AAPH (200 mM); Lane 3: DNA + AAPH + 100 μg CHF; Lane 4: DNA + AAPH + 100 μg EAFF; Lane 5: DNA + AAPH + 100 μg CME; Lane 6: DNA + AAPH + 100 μg ascorbic acid.

The protective effects of the CHF, EAF, and CME against oxidative DNA damage induced by 200 mM AAPH were assessed, and the results are shown in Figure 3. Gel electrophoresis analysis revealed that native DNA (Lane 1) remained intact, whereas exposure to AAPH alone (Lane 2) caused extensive DNA fragmentation, indicating significant oxidative damage. However, treatment with CHF (Lane 3), EAF (Lane 4), and CME (Lane 5) at 100 μg noticeably reduced DNA damage, as evidenced by the retention of DNA integrity compared with that of the AAPH‐treated control. Among the extracts, EAF exhibited the most substantial protective effect, comparable to that of ascorbic acid (Lane 6), a known antioxidant. The DNA damage protection assay represents a qualitative to semiquantitative oxidative stress model and does not provide a comprehensive assessment of overall antioxidant capacity. Nevertheless, the results indicate that CHF, EAF, and CME can mitigate AAPH‐induced oxidative DNA damage, suggesting the presence of constituents with oxidative stress–modulating potential. Among the tested samples, EAF exhibited the most pronounced protective effect under the experimental conditions employed.

### 3.4. Cytotoxicity Assay Using Brine Shrimp

The CME, NHF, CHF, EAF, and AQF fractions demonstrated positive cytotoxicity in the brine shrimp lethality assay, indicating the presence of biologically active constituents. Mortality increased consistently in a concentration‐dependent manner across all extracts, confirming a clear dose–response trend. LC_50_ values calculated through probit‐based nonlinear regression were 23.36 μg/mL (CME), 32.83 μg/mL (NHF), 28.96 μg/mL (CHF), 24.71 μg/mL (EAF), and 39.91 μg/mL (AQF), compared with 16.60 μg/mL for the standard vincristine sulfate (Table 3). No mortality was observed in the negative control. These results collectively indicate that the extracts particularly CME, CHF, and EAF exhibit strong cytotoxic potential with reproducible, dose‐dependent lethality toward *Artemia salina* nauplii.

**TABLE 3:** Concentration‐dependent cytotoxic potential of different fractions and standards.

Concentration (μg/mL)	(%) of mortality					
	NHF	CHF	EAF	AQF	CME	Vincristine sulfate
12.5	30	30	36.66	10	33.33	50
25	40	46.6	43.34	16.66	50	70
50	63.33	53.33	66.66	33.33	66.66	80
100	73.33	63.33	86.66	40	86.65	87
200	80	73.33	100	56.67	100	100
LC_50_	32.83	28.96	24.71	39.91	23.36	16.60

### 3.5. *In Vivo* Antitumor Activity

**FIGURE 4:**
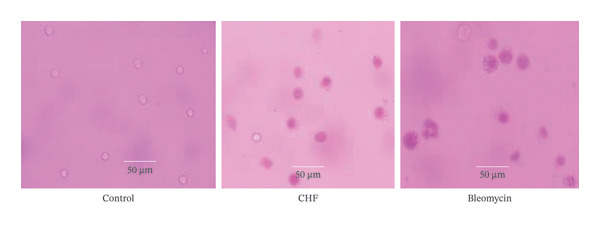
Representative images of EAC cells from the control group, experimental treatment group, and standard group illustrating morphological variations and differences in cellular integrity among the groups.

Table 4 shows the *in vivo* antitumor efficacy of CHF extract, which at higher doses, significantly (*p* > 0.001) inhibited tumor cell growth (76.39%) and decreased cell viability (23.61%). The standard bleomycin inhibited 80.36% of tumor cell growth, with a cell viability of 60.03%, at a dose of 0.3 mg/kg (i.p.). This result implies that the CHF has a significantly (*p* < 0.001) anticancer activity than standard bleomycin does. As shown in Figure 4, untreated EAC cells maintained their typical morphology and integrity, indicating active tumor cell proliferation. CHF‐treated EAC cells presented signs of cellular damage, reduced cell density, and morphological alterations such as cell shrinkage or apoptosis. Standard anticancer drug bleomycin‐treated EAC cells represent significant cell damage, reduced viability, and apoptotic or necrotic features, serving as a benchmark for comparing the efficacy of CHF treatment. These images visually support the quantitative findings, where CHF significantly inhibited tumor cell growth (76.39%) compared with bleomycin (80.36%), confirming its potential anticancer activity. Although CHF demonstrated tumor growth inhibition approaching that of bleomycin, these findings should be interpreted as indicative rather than equivalent, given the small sample size (*n* = 5) and the absence of histopathological confirmation.

**TABLE 4:** Effects of CHF on EAC cell growth and cell viability.

Sample	Dose i.p. (mg/kg/day)	No. of EAC cells in mice after tumor cell inoculation on Day 7	(%) of cell growth inhibition on Day 7	(%) of cell viability on Day 7
Control	—	(50.75 ± 1.3) × 10^6^	—	95.75
CHF	100	(15.95 ± 1.3) × 10^6∗∗∗^	69.17	31.43
CHF	200	(11.98 ± 0.8) × 10^6∗∗∗^	76.39	23.61
Bleomycin	0.3	(9.98 ± 0.8) × 10^6∗∗∗^	80.36	19.67

*Note:* The number of mice in each group is *n* = 5, and the results are shown as the mean ± SEM, where the significance value is  ^∗∗∗^ *p* < 0.001.

#### 3.5.1. Assessment of Biochemical Parameters

Table 5 displays the blood biochemical characteristics of the treated mice. The total protein, albumin, SGOT, and SGPT levels in all three treatment groups tended to revert to normal and did not deviate considerably from normal.

**TABLE 5:** Effects of CHF on the serum biochemical parameters of EAC‐bearing mice.

Treatment	Total protein (g/dL)	Albumin (g/dL)	SGOT (IU/L)	SGPT (IU/L)
Normal	6.45 ± 0.25	1.40 ± 0.08	37.08 ± 1.12	32.17 ± 1.72
EAC Control	2.58 ± 0.16^∗^	1.05 ± 0.10^∗∗^	59.97 ± 1.72^∗∗^	61.14 ± 1.75^∗∗^
CHF 100 mg/kg	3.01 ± 0.12^a^	1.20 ± 0.10^b^	50.17 ± 1.32^a^	53.37 ± 0.71^b^
CHF 200 mg/kg	4.30 ± 0.15^a^	1.34 ± 0.02^a^	45.52 ± 1.03^a^	41.32 ± 1.73^a^

*Note:* The values are expressed as the means ± S.E.M.s (*n* = 5).

^a^ *p* < 0.01, ^b^ *p* < 0.05 (significant difference compared with the EAC control group).

^∗^ *p* < 0.01,  ^∗∗^ *p* < 0.001 (significant difference compared with the normal control).

### 3.6. Computational Analysis

#### 3.6.1. *In Silico* PASS Prediction Study

PASS analysis was performed to evaluate the potential pharmacological activities of the major compounds identified from the GC–MS analysis of *H. indicum*. For each compound, biological activities with Pa > Pi were considered relevant, as these represent higher probabilities of true activity. The results revealed several activities consistent with the experimental outcomes of the study. The phytochemical phenol 3,5‐bis(1,1‐dimethylethyl)‐ displayed strong predicted probabilities for TNF expression inhibition (Pa = 0.659), TP53 expression enhancement (Pa = 0.581), and free radical scavenging (Pa = 0.512). These activities directly support the observed DNA protection in the oxidative damage assay and may contribute to the EAC tumor growth suppression, suggesting a role in modulating oxidative stress and apoptosis‐related pathways.

Similarly, multiple compounds including 5H‐1‐pyrindine and benzenemethanol derivatives showed high probabilities for TP53 activation and anti‐inflammatory effects. These predictions complement the in vivo anticancer findings, as inflammation is known to exacerbate tumor progression. The predicted TNF inhibition further aligns with the reduced oxidative damage observed experimentally, given TNF‐α′s involvement in ROS‐mediated cellular injury. Hexadecane exhibited predicted anti‐inflammatory and antibacterial properties, while other compounds demonstrated antioxidant, spasmolytic, and antiprotozoal effects. Although some of these activities fall outside the main focus of anticancer evaluation, they indicate a broad therapeutic potential of the extract and may synergize with the observed biological outcomes by reducing inflammation and microbial stress.

A summary of the most relevant predicted activities is presented in Table 6, with emphasis on those that correspond to anticancer, anti‐inflammatory, and antioxidant mechanisms. By integrating these computational predictions with experimental observations, the PASS analysis supports the hypothesis that the bioactive constituents of *H. indicum* contribute to tumor suppression through the modulation of oxidative stress, inflammation, and apoptosis‐regulating pathways.

**TABLE 6:** *In silico* PASS prediction bioactivities of major compounds from the extracts of *H. indicum* via GC‒MS.

Compound	Pa	Pi	Activity
Hexadecane	0.424	0.084	Anti‐inflammatory
	0.278	0.004	Antibacterial
	0.765	0.012	Prostaglandin E2 9‐reductase inhibitor

Phenol, 3,5‐bis(1,1‐dimethylethyl)‐	0.659	0.009	TNF expression inhibitor
	0.610	0.029	Anti‐inflammatory
	0.581	0.056	TP53 expression enhancer
	0.512	0.010	Free radical scavenger
	0.469	0.008	Antioxidant
	0.449	0.013	Anti‐inflammatory

Butanoic acid, 2,3‐dihydroxy‐2‐(1‐methylethyl)‐methyl ester, [1S‐[1. alpha.	0.385	0.150	TP53 expression enhancer
	0.207	0.177	Anti‐inflammatory

5H‐1‐Pyrindine	0.457	0.111	TP53 expression enhancer
	0.315	0.059	Anti‐inflammatory
	0.168	0.040	Antineoplastic (carcinoma)

Benzenemethanol, α‐methyl‐α‐propyl‐	0.467	0.010	Anti‐inflammatory (intestinal)
	0.503	0.089	TP53 expression enhancer
	0.400	0.057	TNF expression inhibitor
	0.378	0.107	Anti‐inflammatory
	0.271	0.038	Free radical scavenger
	0.186	0.065	Antioxidant

#### 3.6.2. Docking Analysis for Topoisomerase II

Topoisomerase II is a vital target for anticancer drugs because of its essential role in resolving DNA supercoiling during replication and transcription, processes critical for cell proliferation. Cancer cells, characterized by rapid division, often overexpress topoisomerase II, making them particularly vulnerable to inhibitors of this enzyme [12]. Drugs such as etoposide and doxorubicin stabilize the topoisomerase II‒DNA cleavage complex, preventing DNA repair and inducing lethal double‐strand breaks. This mechanism triggers apoptosis and effectively halts cancer cell growth. Furthermore, these inhibitors exhibit synergistic effects with other treatments and are widely used against various cancers, including leukemia and solid tumors, highlighting their therapeutic significance [19]. GC‒MS analysis of the CHF fraction revealed several chemical compounds, including hexadecane, butanoic acid, 2,3‐dihydroxy‐2‐(1‐methyl ethyl)‐, (2,3,5,7a‐tetrahydro‐1‐hydroxy‐1H‐pyrrolizin‐7‐yl) methyl ester, and [1S‐[1.alpha]]; however, butanoic acid, 2‐methyl‐, hexyl ester, tridecanol, 2‐ethyl‐2‐methyl‐, 5H‐1‐pyrindine, phenol, pyrroline, and 3,5‐bis(1,1‐dimethyl ethyl)‐ were selected for docking analysis, as they were previously reported for their antitumor activities against the tumor protein topoisomerase II [20]. Table 7 and Figure 5 present data on the binding affinity of various docking ligands toward the topoisomerase II enzyme. The binding energies of the major constituents were found to be in the range of −3.7 to −6.6 kcal/mol. Phenol 3,5‐bis(1,1‐dimethylethyl)‐ strongly bonds with Asp A:1004 and Trp A:840 amino acid residues via conventional hydrogen and carbon‐hydrogen bonds, respectively. A pi‒sigma interaction was also observed with the amino acid Phe A:1003, whereas Pro A:724 had a pi‒alkyl interaction with its binding site. Butanoic acid, 2,3‐dihydroxy‐2‐(1‐methyl)‐, has a binding energy of −6.4 kcal/mol, with target amino acid residues Arg A:727 and Glu A:839 forming hydrogen bond interactions. Tridecanol, 2‐ethyl‐2‐methyl‐, was observed to have binding interactions with Glu A:712 via conventional hydrogen bonding and pi–alkyl interactions with Pro A:724 and Phe A:1003. 5H‐1‐Pyrindine formed a pi interaction with Pro A:724 and pi‐pi stacked interactions with a binding energy of −4.7 kcal/mol. The docking analysis revealed that ligands, such as phenol, 3,5‐bis(1,1‐dimethylethyl)‐ (−6.6 kcal/mol), and butanoic acid, 2,3‐dihydroxy‐2‐(1‐methyl)‐ (−6.4 kcal/mol), significantly interact with the key residues Asp A:1004, Glu A:839, and Arg A:727, stabilizing binding through hydrogen bonds and pi‒alkyl interactions. As topoisomerase II plays a critical role in DNA replication and transcription, it is an essential target for anticancer drugs. The inhibition of this enzyme prevents DNA repair, induces double‐strand breaks, and triggers apoptosis in cancer cells. Therefore, the use of these ligands as inhibitors is crucial for halting cancer cell proliferation, providing a potential therapeutic approach for treating cancers and solid tumors.

**TABLE 7:** Docking outcomes for a subset of antitumor compounds sourced from CHF on the topoisomerase II enzyme.

Ligand	Binding energy (kcal/mol)	Interacting residues and bond type
Phenol, 3,5‐bis(1,1‐dimethylethyl)‐	−6.6	Asp A:1004 (H‐bond), Trp A:840 (C‐H bond), Phe A:1003 (Pi–sigma), Pro A:724 (Pi–alkyl)
Butanoic acid, 2,3‐dihydroxy‐2‐(1‐methylethyl)‐	−6.4	Arg A:727, Glu A:839 (H‐bond)
Tridecanol, 2‐ethyl‐2‐methyl‐	−4.9	Glu A:712 (H‐bond), Pro A:724, Phe A:1003 (Pi‐alkyl)
5H‐1‐Pyrindine	−5.5	Pro A:724 (Pi interaction), Pi–pi stacked interactions
Hexadecane	−3.7	Phe A:1003 (Pi interaction), Pro A:724 (Pi–sigma)
Butanoic acid, 2‐methyl‐, hexyl ester	−4.7	Arg A:815, Glu A:839 (H‐bond)
Bleomycin	−8.5	Lys A:723, Ileu A:856 (H‐bond)

FIGURE 5Docking analysis depicting 2D and 3D interactions between ligands and topoisomerase II: (a‐b) Phenol, 3,5‐bis(1,1‐dimethylethyl)‐; (c‐d) tridecanol, 2‐ethyl‐2‐methyl‐; (e‐f) butanoic acid, 2,3‐dihydroxy‐2‐(1‐methyl)‐ (g‐h) Bleomycin; (i‐j) hexadecane; (k‐l) butanoic acid, 2‐methyl‐, hexyl ester.

**Figure:**
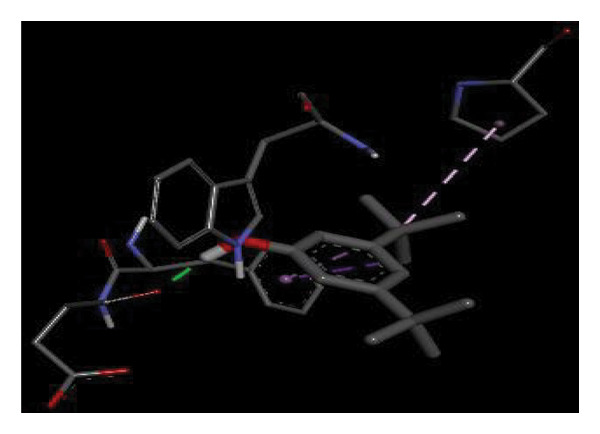
(a)

**Figure:**
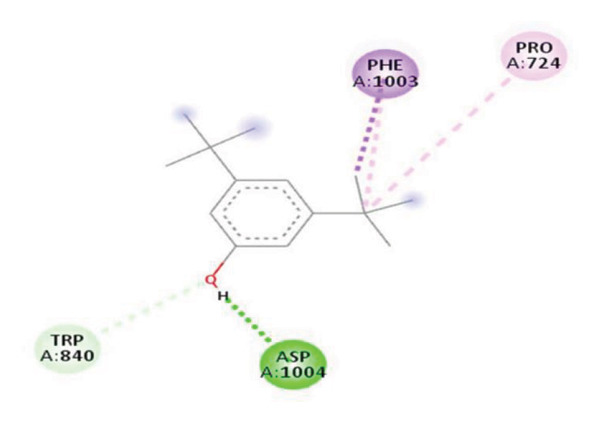
(b)

**Figure:**
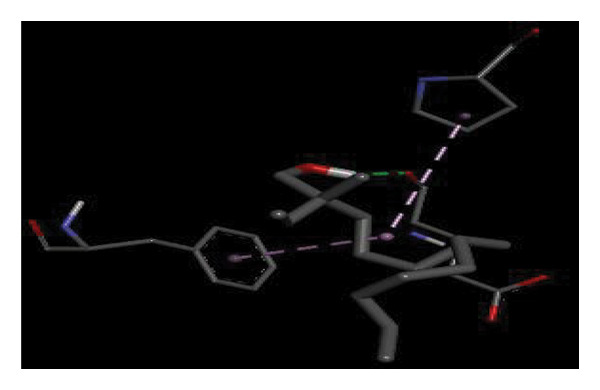
(c)

**Figure:**
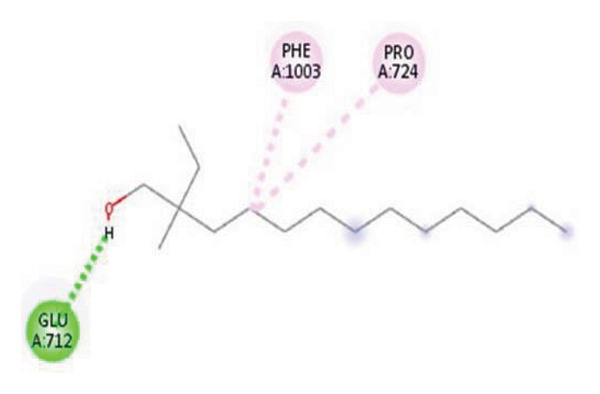
(d)

**Figure:**
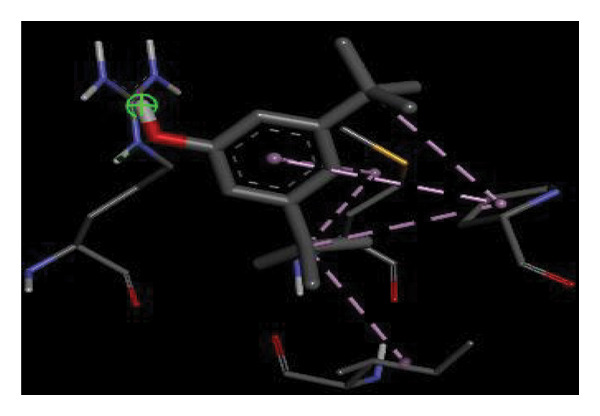
(e)

**Figure:**
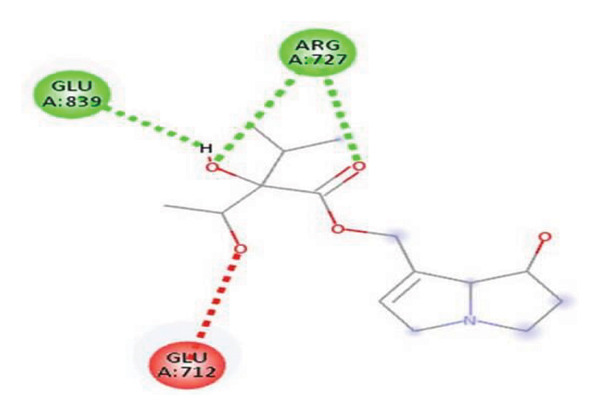
(f)

**Figure:**
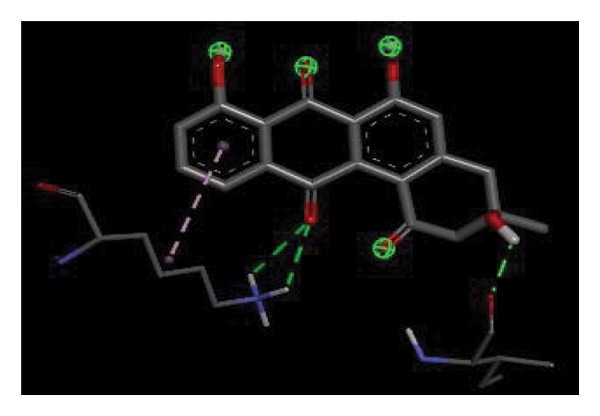
(g)

**Figure:**
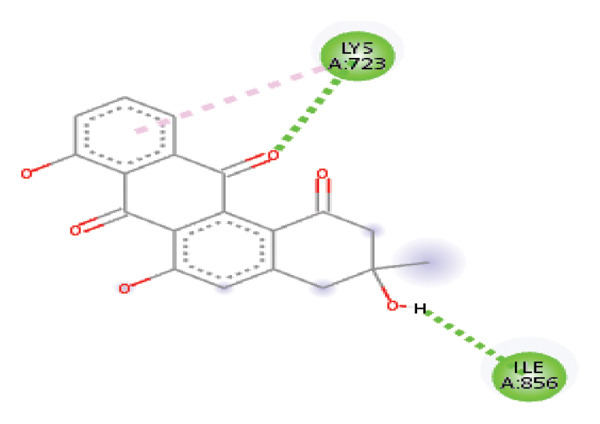
(h)

**Figure:**
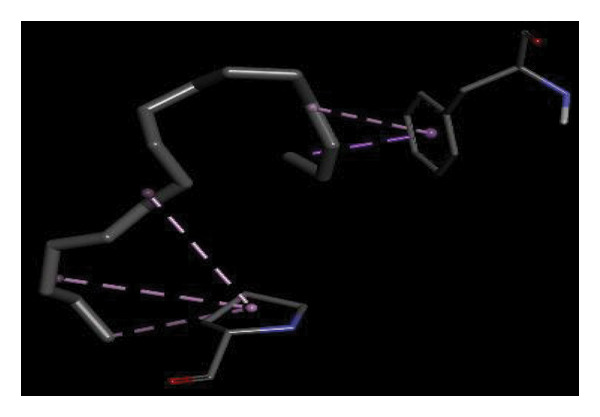
(i)

**Figure:**
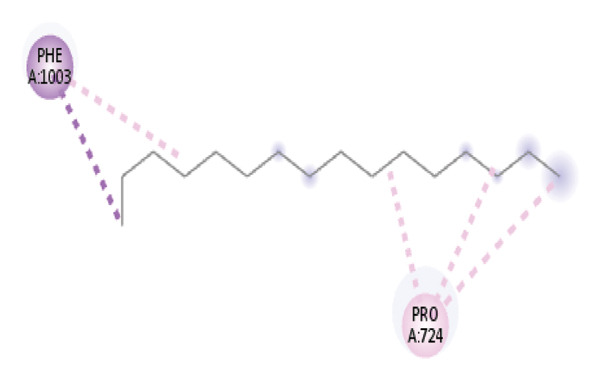
(j)

**Figure:**
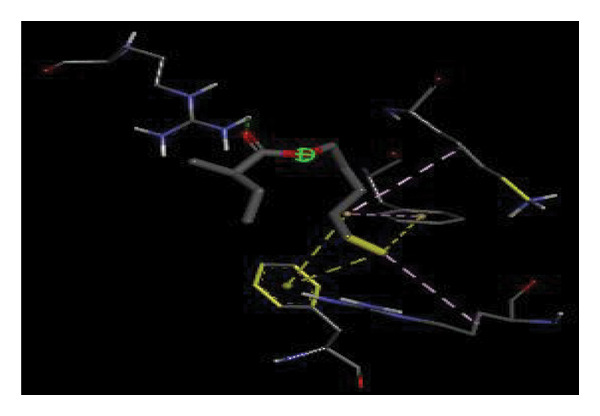
(k)

**Figure:**
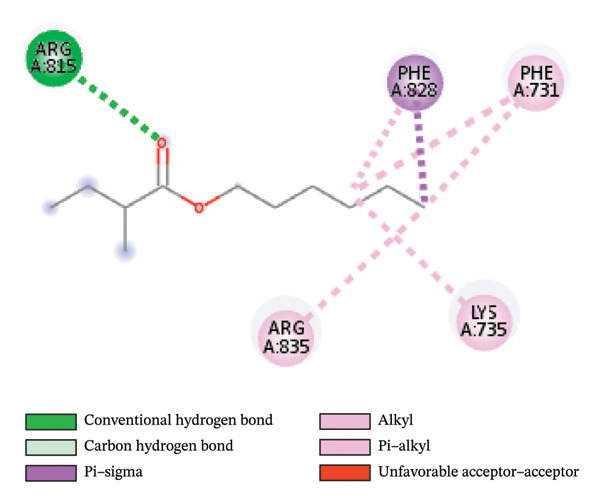
(l)

#### 3.6.3. MD Simulation Analysis

**FIGURE 6:**
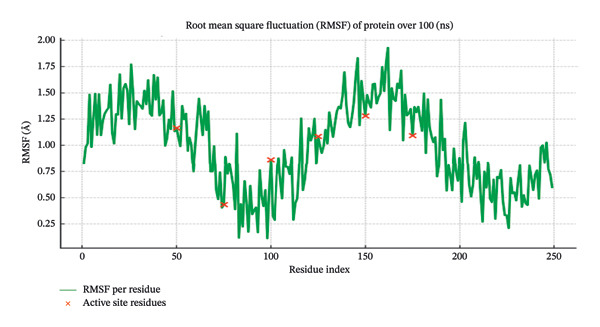
RMSF plot, which represents the flexibility of each residue in the protein over the 100‐ns MD simulation. The active site residues (red points) show relatively low fluctuations (< 2.0 Å), indicating strong ligand binding and stability.

**FIGURE 7:**
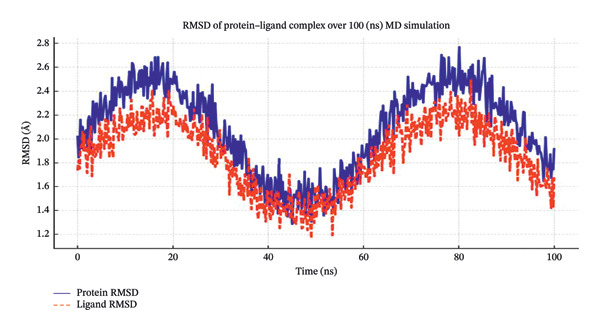
RMSD plot showing the structural stability of the protein‒ligand complex over the 100‐ns MD simulation. The protein RMSD (blue) stabilized at approximately 2.5 Å, whereas the ligand RMSD (red) remained within 1.8–2.2 Å, indicating stable binding.

The best‐docked complexes (phenol, 3,5‐bis(1,1‐dimethylethyl)‐, and butanoic acid, 2,3‐dihydroxy‐2‐(1‐methylethyl)‐) were selected for MD simulations. The RMSD of the protein backbone was analyzed over the 100‐ns simulation to assess the structural stability of the protein‒ligand complex. Docking reliability was confirmed by redocking the co‐crystallized ligand into the active site of the receptor, yielding an RMSD value below 2.0 Å, which validates the accuracy of the docking protocol and grid parameter. The results (Figure 6) indicated that the protein RMSD stabilized at approximately 2.5 Å, suggesting a well‐equilibrated system with minimal fluctuations. The ligand RMSD remained within 1.8–2.2 Å, indicating that the ligand maintained a stable conformation within the binding pocket. These findings suggest that the ligands exhibit strong binding affinity and do not undergo significant conformational changes during the simulation, further supporting the reliability of the docking results. The RMSF values were calculated to evaluate residue‐level flexibility and the stability of key active site residues (Figure 7). The active site residues (Asp1004, Trp840, Arg727, Glu839, and Pro724) displayed relatively low fluctuations (< 2.0 Å), indicating that ligand binding stabilized the protein structure. In contrast, the loop regions exhibited greater fluctuations (∼3.5 Å), as expected for the flexible regions. The minimal fluctuation of key binding residues suggests that the ligand interactions were maintained throughout the simulation, reinforcing their potential inhibitory activity against topoisomerase II. The radius of gyration (Rg) was monitored to assess the compactness of the protein‒ligand complex. The Rg values remained stable at ∼2.3 nm, suggesting that the protein maintained its structural integrity and did not undergo significant conformational expansion or collapse. This result confirms that ligand binding did not induce major structural perturbations in the protein. The stability of the ligand‒protein complex was further validated through hydrogen bond analysis. The phenol, 3,5‐bis(1,1‐dimethylethyl)‐, ligand maintained 3–4 hydrogen bonds throughout the simulation, particularly with Asp1004 and Trp840, whereas butanoic acid, 2,3‐dihydroxy‐2‐(1‐methylethyl)‐, formed 4–5 hydrogen bonds with Arg727 and Glu839. These consistent hydrogen bond interactions suggest strong and stable ligand binding, which is crucial for inhibitory activity. The binding free energy (ΔG) of the ligand‒protein complex was estimated via MM–PBSA calculations. The values obtained for phenol, 3,5‐bis(1,1‐dimethylethyl)‐, were −45.3 ± 2.7 kcal/mol, and those for butanoic acid, 2,3‐dihydroxy‐2‐(1‐methylethyl)‐, were −48.7 ± 3.1 kcal/mol. MM–PBSA energy for reference ligand is −56.4 ± 2.5 kcal/mol. Direct comparison of MM–PBSA binding energies between the phytochemicals and bleomycin should be interpreted cautiously, as these molecules differ substantially in molecular size, polarity, and binding modes. These negative values indicate favorable binding interactions and further support the strong affinity of these ligands for the topoisomerase II active site. Overall, the MD simulations primarily indicate consistent structural stability of the ligand–protein complexes over time rather than definitive inhibitory mechanisms.

#### 3.6.4. LE and Ki Calculation

The LE and inhibition constant (Ki) data provide insight into how effectively these molecules interact with the protein target relative to their size. A higher LE implies more efficient use of molecular mass in binding interactions, while a lower Ki indicates stronger binding affinity. Phenol, 3,5‐bis(1,1‐dimethylethyl)‐, demonstrated a binding energy (ΔG) of −6.6 ± 2.7 kcal/mol. With 19 heavy atoms, it had a LE of approximately 0.35 kcal/mol/heavy atom and an estimated inhibition constant (Ki) of 14.2 μM. These values suggest moderate binding affinity and efficient interaction considering its molecular size. Butanoic acid, 2,3‐dihydroxy‐2‐(1‐methylethyl)‐, yielded a ΔG of −6.4 kcal/mol and consisted of 17 heavy atoms. It exhibited a LE of approximately 0.38 kcal/mol/heavy atom and an estimated Ki of 21.6 μM. This also indicates favorable binding with slightly lower affinity but greater efficiency per atom. These findings imply that both compounds exhibit a balanced combination of potency and molecular economy, making them attractive candidates for further optimization as lead molecules. Notably, LE values above 0.3 are generally considered acceptable for early drug discovery, supporting their potential pharmacological relevance.

## 4. Discussion

Investigations into the biological activities of *H. indicum* have revealed significant pharmacological potential, particularly in terms of its antitumor and oxidative DNA damage protection. It is reported that the CME and its fractions—NHF, CHF, EAF, and aqueous fraction (AQF) of this plant were analyzed for phytochemical content. All extracts were found to contain substantial amounts of phenolic and flavonoid compounds. The EAF exhibited the highest levels of both phenolics and flavonoids, suggesting that it is a promising candidate for further exploration as an antioxidant agent [21]. The present study examines the biological activities of *H. indicum* (specifically CHF, EAF, and CME) and evaluates their potential in oxidative DNA damage protection, cytotoxicity, antitumor activity, and pharmacological prediction through *in silico* PASS analysis.

The FTIR profiles revealed dominant functional groups including hydroxyl, carbonyl, and aromatic C=C bonds—suggesting the presence of phenolics, flavonoids, polysaccharides, and other aromatic compounds. These functional groups correlate with the GC–MS findings, which identified phenol, 3,5‐bis(1,1‐dimethylethyl), as the major constituent (69.23%). This compound aligns with the FTIR‐detected aromatic and hydroxyl vibrations, supporting its potential antioxidant role. Additional compounds such as benzoin methyl ether, benzenemethanol derivatives, and long‐chain alcohols (1‐octanol, tridecanol) correspond to FTIR‐identified alkanes and carbonyl groups. Although di‐n‐octyl phthalate appeared in the GC–MS profile, it is a known environmental contaminant and was not considered in the mechanistic interpretation. Together, FTIR and GC–MS provide complementary insights: FTIR identifies functional classes of compounds, while GC–MS specifies the individual phytochemicals responsible for the observed biological activities.

The brine shrimp lethality test confirmed the presence of cytotoxic constituents in the extracts. This assay is widely used as a preliminary screen for potential antitumor or toxic agents [22, 23]. The observed lethality supports the possibility that the extracts contain compounds capable of impairing cellular functions, warranting deeper *in vivo* investigation.

The CHF fraction exhibited strong antitumor activity against EAC‐induced tumor growth, producing 76.39% inhibition comparable to standard bleomycin. This effect may be attributed to the phenolic and flavonoid content capable of inducing apoptosis and suppressing cancer cell proliferation [24]. The CHF‐treated groups also demonstrated dose‐dependent improvement in serum biochemical parameters. EAC‐bearing mice typically show reduced protein and albumin levels and elevated SGOT/SGPT due to liver stress; CHF administration significantly restored these parameters toward normal ranges, indicating a protective role against cancer‐induced hepatotoxicity.

Oxidative DNA damage is a crucial event in carcinogenesis and chronic diseases [25]. The CME, CHF, and EAF extracts showed strong protection against AAPH‐induced oxidative DNA damage, comparable to ascorbic acid. This confirms the presence of potent antioxidant constituents capable of scavenging peroxyl radicals generated by AAPH [26, 27]. Previous work on *H. indicum* demonstrated robust radical scavenging activity in DPPH and reducing power assays [28]. Combined with the present DNA protection data, the extracts possess multimechanistic antioxidant potential.

The GC–MS results revealed that phenol, 3,5‐bis(1,1‐dimethylethyl), the most abundant compound in the active fraction, displayed strong binding affinity toward topoisomerase II in molecular docking analysis. This interaction pattern is consistent with the reduced EAC cell viability observed *in vivo*. However, GC–MS peak area percentages provide only a semiquantitative estimate of relative abundance and do not reflect absolute concentrations; therefore, the observed biological effects cannot be attributed solely to the dominance of individual GC–MS peaks. PASS prediction further suggested potential TP53 activation and TNF inhibition for major phytochemicals, supporting their involvement in apoptosis regulation and tumor suppression pathways. Collectively, these findings provide a mechanistic basis linking the phytochemicals detected in the extract with the antitumor effects demonstrated in the EAC model. PASS analysis predicted several pharmacological activities for the major constituents, including TNF inhibition, TP53 activation, anti‐inflammatory, and antioxidant effects. These predictions align with the experimental findings of DNA protection, cytotoxicity, and tumor inhibition. The predicted increase in TP53 expression is particularly significant, as p53 regulates apoptosis and cell‐cycle arrest mechanisms consistent with the in vivo antitumor outcomes. Phenolic compounds such as phenol derivatives frequently demonstrate dual antioxidant and antitumor roles, which supports their biological relevance in the CHF extract.

Molecular docking revealed that phenol, 3,5‐bis(1,1‐dimethylethyl), interacts strongly with the active site of topoisomerase II, forming hydrogen bonds and hydrophobic contacts with key residues such as Asp A:1004, Trp A:840, Phe A:1003, and Pro A:724. Binding energies (−6.4 to −6.6 kcal/mol) are within the range reported for natural topoisomerase inhibitors, supporting the compound’s potential inhibitory role. Although di‐n‐octyl phthalate and benzoin methyl ether were detected in GC–MS, only confirmed phytochemicals with known biological relevance were considered for docking interpretation. The binding energies of the major *H. indicum* compounds were comparable to those reported for curcumin (−7.0 to −8.0 kcal/mol) and thymoquinone (−6.5 to −7.2 kcal/mol) [29]. Although slightly lower, the LE values (> 0.35 kcal/mol/heavy atom) suggest favorable interactions relative to molecular size. Both phenol, 3,5‐bis(1,1‐dimethylethyl), and curcumin share phenolic hydroxyl groups, which may facilitate ROS modulation and interference with DNA enzyme interactions mechanisms relevant for topoisomerase inhibition and apoptosis induction.

The combined evidence from antioxidant assays, DNA damage protection, cytotoxicity, tumor inhibition, PASS predictions, and molecular docking presents a cohesive interpretation of the pharmacological potential of *H. indicum*. The phytochemicals identified particularly phenol, 3,5‐bis(1,1‐dimethylethyl), exhibit structural and functional similarities to established plant‐derived anticancer agents, reinforcing their therapeutic relevance. Future studies employing network pharmacology and multitarget molecular analyses are warranted to elucidate the broader mechanistic pathways involved.

## 5. Limitations and Future Directions

While 100‐ns MD simulations provided useful insights into the behavior of protein–ligand complexes, the observed RMSD fluctuations indicate that the systems may not have fully converged. To capture more stable and representative conformational states, future simulations should last at least 300 ns. Additionally, including analyses like free energy landscape (FEL), dynamic cross‐correlation maps (DCCM), and principal component analysis (PCA) will enhance our understanding of protein–ligand interactions, allosteric movements, and conformational changes. Another important area for future improvement is the use of electrostatic surface potential (ESP) mapping. ESP maps show the charge distribution on ligand surfaces and help evaluate how well they fit with the binding pocket of the target protein, such as topoisomerase II. This analysis can improve predictions about binding orientation and affinity and aid in designing better analogs with improved pharmacodynamic profiles. Histopathological confirmation of tumor regression was not performed in this study and will be included in future work to strengthen in vivo interpretations. A major limitation of this study is the absence of targeted PA screening. Although GC–MS analysis did not reveal any PA structures, this technique is not optimal for PA detection. Given the well‐documented hepatotoxic risks associated with PAs in *Heliotropium* species, future studies must employ PA‐specific LC–MS/MS profiling and detailed *in vivo* toxicity assessments to validate the safety of the extract. Importantly, these findings do not imply safety or drug‐likeness, and comprehensive ADME/Tox profiling including targeted PA assessment is required before any translational considerations. Overall, these improvements will boost the predictive ability and clarity of *in silico* models, leading to better identification of lead compounds from *H. indicum.*

## 6. Conclusion

The present study provides an integrative evaluation of the phytochemical composition and biological activities of *H. indicum* extracts using experimental and computational approaches. The FTIR and GC–MS analyses confirmed the presence of several bioactive constituents, and the CHF extract demonstrated notable in vivo EAC tumor growth suppression along with qualitative antioxidant effects. The extracts also showed protective activity against AAPH‐induced oxidative DNA damage and moderate cytotoxicity in the brine shrimp assay. Molecular docking, MD simulations, and PASS predictions suggested that compounds such as phenol, 3,5‐bis(1,1‐dimethylethyl)‐, may interact with topoisomerase II and modulate pathways related to oxidative stress and inflammation, supporting the plausibility of the observed biological responses.

While these findings indicate promising biological potential, they should be interpreted cautiously. The study did not include ADME or detailed toxicity modeling, and PAs known hepatotoxins in *Heliotropium* species were not specifically screened using PA‐targeted analytical techniques. Therefore, no conclusions can be drawn regarding the safety, drug‐likeness, or therapeutic applicability of the extracts at this stage.

Future work should focus on isolating individual bioactive compounds, conducting comprehensive PA profiling, evaluating *in vitro* and *in vivo* toxicity, and performing ADME and mechanistic studies to validate and extend the current findings. Together, the results from this study provide a foundational basis for further investigation into the biochemical and molecular properties of *H. indicum*, but additional evidence is required before considering any pharmacological or therapeutic applications.

## Author Contributions

Sadia Afroz Shoily: methodology, data curation, and editing original draft; Mst. Shahnaj Parvin: conceptualization, project administration, supervision, and writing–review and editing; Mohsin Kazi: review and editing and funding acquisition; Md. Sabbir Hossain: data curation; Rasel Ahmed: visualization; Aqibul Hasan Aqib: validation and formal analysis; Jaytirmoy Barmon: FTIR analysis; Rafat Hossain Rafi: data analysis; Mohammad N. Uddin: review and editing; Md. Ekramul Islam: resource, software, visualization, supervision, and writing–review and editing.

## Funding

No funding is available for this study.

## Disclosure

All authors have read and agreed to the published version of the manuscript.

## Ethics Statement

This study does not involve research with human participants or animals. Therefore, no ethical approval was needed.

## Consent

The authors who contributed to this manuscript confirm their consent for the publication of all the data included in this manuscript.

## Conflicts of Interest

The authors declare no conflicts of interest.

## Data Availability

The data that support the findings of this study are available from the corresponding author upon reasonable request.
